# Adverse Events and Drug Interactions Associated with Elexacaftor/Tezacaftor/Ivacaftor Treatment: A Descriptive Study Across Australian, Canadian, and American Adverse Event Databases

**DOI:** 10.3390/life15081256

**Published:** 2025-08-07

**Authors:** Theeba Thiruchelvam, Chiao Xin Lim, Courtney Munro, Vincent Chan, Geshani Jayasuria, Kingsley P. Coulthard, Peter A. B. Wark, Vijayaprakash Suppiah

**Affiliations:** 1 Discipline of Pharmacy, School of Health and Biomedical Sciences, RMIT University, Bundoora, VIC 3083, Australia; theeba.thiruchelvam1@health.nsw.gov.au (T.T.); chiao.xin.lim@rmit.edu.au (C.X.L.); vincent.chan@rmit.edu.au (V.C.); 2 John Hunter Hospital, Newcastle, NSW 2305, Australia; geshani.jayasuriya@health.nsw.gov.au; 3 Medicine Department, School of Clinical Sciences, Monash University, Clayton, VIC 3168, Australia; 4 Murdoch Children’s Research Institute, Parkville, VIC 3010, Australia; courtney.munro@mcri.edu.au; 5 Department of Pediatric and Adolescent Gynaecology, The Royal Children’s Hospital Melbourne, Parkville, VIC 3010, Australia; 6 Department of Pediatrics, University of Melbourne, Melbourne, VIC 3004, Australia; 7 Clinical and Health Sciences, University of South Australia, Adelaide, SA 5000, Australia; kingsleycoulthard@gmail.com; 8 School of Translational Medicine, Monash University, Melbourne, VIC 3004, Australia; peter.wark@monash.edu; 9 Respiratory and Sleep Medicine, Alfred Health, Melbourne, VIC 3004, Australia; 10 Australian Centre for Precision Health, University of South Australia, Adelaide, SA 5000, Australia

**Keywords:** adverse events, drug–drug interactions, cystic fibrosis, elexacaftor/tezacaftor/ivacaftor

## Abstract

People with cystic fibrosis may experience polypharmacy, which can increase the risk of drug induced complications such as adverse events and drug–drug interactions. This study aimed to examine the prevalence of adverse events and to identify potential drug–drug interactions associated with elexacaftor/tezacaftor/ivacaftor (ETI). Three databases, the Australian Therapeutic Goods Administration Database of Adverse Event Notification (TGA DAEN), the Canada Vigilance Adverse Reaction Online Database (CVAROD), and the USA Food and Drug Administration Adverse Event Reporting System (FAERS) Database were searched for spontaneous ETI adverse events between 2019 and 2024. Descriptive analysis of the data was undertaken. The FAERS database was analysed to identify adverse events of interest such as anxiety and depression and concomitant drugs prescribed with ETI. A total of 10,628 ETI associated adverse events were identified in all system organ classes. The incidence of psychiatric adverse events ranged from 7 to 15% across the three databases. Potential drug–drug interactions with CYP 3A4/5 strong inhibitors and strong inducers were identified from the FAERS database and azole antifungals were implicated in several ETI dose modifications. The prevalence and types of ETI adverse events were varied and use of concomitant drugs with potential drug interactions was significant, requiring more research to manage them.

## 1. Introduction

Cystic fibrosis (CF) is a complex multiorgan autosomal recessive disease caused by genetic variants in the cystic fibrosis transmembrane conductance regulator (CFTR) gene. This results in a decrease in the quantity and/or activity of the CFTR protein [1]. CFTR modulators are the mainstay treatment for CF where they improve the production, intracellular processing, and function of the CFTR protein [2]. The triple combination of CFTR modulators elexacaftor/tezacaftor/ivacaftor (ETI) was registered with the U.S. Food and Drug Administration (FDA) in 2019 for people with CF who carry at least one copy of the variant F508del. Clinical trials demonstrated that treatment with ETI significantly improved lung function, with percentage predicted forced expiratory volume in one second (ppFEV1) increasing by up to 14%, along with reductions in lung exacerbations and improvements in quality of life of people with CF [3,4].

People with CF often have multiple comorbidities, including diabetes (up to 35% of adults), anxiety (22–32%), and depression (10–19%) [5]. Therefore, polypharmacy, defined as five or more medications, is common in people with CF. This increases the risk of drug-induced complications, including adverse events and drug–drug interactions [6,7]. Advances in CF care have increased the life expectancy of people with CF to over 50 years of age, and this may increase further with extensive use and early introduction of CFTR modulators [8]. The increase in life expectancy for people with CF may correlate with co-morbidities that may increase medication burden. Thus, awareness of adverse events and drug–drug interactions is critical in preventing drug-induced toxicities in people with CF as they age [7].

An adverse event is an unexpected and unplanned response to a drug that occurs at normal doses. Clinical trials investigating ETI treatment have reported an acceptable safety profile. However, these trials are often not a true representation of patients seen in clinics, as they tend to be conducted in a small, selected group of participants observed over short study periods. As a result, clinical trials are often underpowered to detect adverse events, particularly those with delayed onset or lower frequency [9]. Several systematic reviews have described the efficacy and safety of CFTR modulators [10,11,12]. Systematic reviews are useful for identifying common and well-documented adverse events. However, they may not capture uncommon and rare adverse events. Small studies have described the wide range of adverse events observed with the use of CFTR modulators in clinical care, but these are in a selected population and maybe subject to reporting bias [13,14]. A recent study by Papadakis et al. reported heterogeneity of adverse events, highlighting the need for close monitoring and timely management of adverse events [15]. The study also found that mental health changes were one of the most reported adverse events, which were also identified in several other studies. Together, these studies highlighted the need for more research on the prevalence of psychiatric adverse events, in particular anxiety and depression, with ETI treatment, which are rarely reported during clinical trials [15,16,17].

Post-marketing surveillance is an important component of pharmacovigilance. International online databases have been used to investigate the prevalence of ‘real world’ adverse events. The Australian Therapeutic Goods Administration Database of Adverse Event Notification (TGA DAEN), the Canada Vigilance Adverse Reaction Online Database (CVAROD), and the United States Food and Drug Administration Adverse Event Reporting System (FAERS) Database are examples of databases that curate adverse drug events reported by healthcare professionals, consumers, and pharmaceutical companies within their respective countries [18,19,20]. Data from these international databases can be analysed to determine the prevalence, severity, risks, and trends of adverse events, as well as patient characteristics and treatment results. This information has been vital in gathering evidence to formulate strategies to mitigate adverse events [21].

Adverse events may result from drug–drug interactions, where one drug affects the efficacy or toxicity of another due to potentially significant changes to drug exposure. Previous studies have highlighted potential drug–drug interactions in individuals taking ETI; however, the clinical relevance of some of these interactions remains unclear [22]. Elexacaftor, tezacaftor, ivacaftor are extensively metabolised by enzymes of cytochrome P450 3A (CYP3A), including enzymes CYP3A4 and CYP3A5 [22,23]. Therefore, serum concentrations of these CFTR modulators can be potentially impacted by patients’ metaboliser phenotype, as well as co-administration of CYP3A4/5 inducers and inhibitors. Ivacaftor is an inhibitor of CYP2C9 and drugs that are substrates of this enzyme (e.g., warfarin) may have their serum concentrations increased, potentially leading to increased rates and severity of adverse events [22]. Also, ETI are known to be substrates and/or inhibitors of certain transport proteins. However, the clinical impact of these interactions is still being elucidated and often less well characterised in comparison to metabolic interactions. Furthermore, the impact of metabolic drug interactions on patient outcomes has not been extensively described in the literature. Hence, analysis of concurrent medications taken by people with CF through these databases may provide some useful information on drug interactions and safety in a real-world setting.

## 2. Aim

The aims of this study were to examine the prevalence of adverse events and identify potential drug–drug interactions associated with ETI as reported in three international adverse events databases.

## 3. Ethics Approval

Ethical approval was not required for this study as it did not involve human participants, animals, or the collection of personal data. Exemption was granted by the ethics committee of RMIT University.

## 4. Methods

### 4.1. Study Design and Data Sources

The Australian, Canadian, and U.S. databases were searched for spontaneous adverse events reported over 4.5 years (last quarter of 2019 to the first quarter of 2024) as ETI was approved by FDA in the latter half of 2019. Adverse events have been classified by body system or organ class according to the Medical Dictionary for Regulatory Activities (MEDRA) which allowed for grouping of adverse events [24].

The U.S. FAERS database was also analysed for concomitant drugs that may be substrates, inhibitors, or inducers of CYP3A4/5 enzymes, and drugs that are substrates of CYP2C9 to investigate the potential for drug–drug interactions and the risk of adverse events.

### 4.2. Search Strategy

All three databases were searched for all adverse events reported for ETI. This included demographic data from the patient (e.g., gender and age). All three databases use the MEDRA System Organ Class (SOC) which enabled a comparison of the adverse events of SOC across the databases. Furthermore, a more detailed analysis of depression and anxiety was performed using the preferred term of the adverse events within the SOC.

Concomitant drugs were identified from the FAERS database because there were a significant number of reports in this database that included concomitant drugs. OpenVigil, which is a tool for the mining and analysis of FAERS database pharmacovigilance data, was used to identify adverse events of interest, in particular anxiety and depression and the concomitant drugs used [25].

### 4.3. Data Analysis

The three datasets differed in the number of reports as the number of people with CF prescribed ETI differed across the three countries. The adverse events reports including patient demographics were exported to Microsoft Excel and descriptive analyses of the data were performed. The number of reports for each SOC was determined and analysed as a proportion of all adverse-event reports for each dataset. The findings utilising percentages were compared across the datasets to determine commonalities and differences. The SOCs with most reports were further analysed to determine the prevalence of specific adverse events. The top three adverse events were determined in the top 14 SOCs for each database.

The details of concomitant drugs, obtained from the FAERS database, were classified as inhibitors or inducers of CYP3A4/5 enzymes and substrates of CYP2C9 using the Flockhart CYP 450 drug–drug interaction table, which listed 40 CYP 3A4/5 inhibitors and 27 CYP 3A4/5 inducers that may have clinically relevant interactions [26]. These inhibitors and inducers were further classified as strong, moderate, or weak. The CYP3A4/5 inhibitors that interact with ETI included itraconazole, voriconazole, and clarithromycin, which are classified as strong inhibitors, while fluconazole and erythromycin are moderate inhibitors. The CYP 3A4/5 inducers which interact with ETI included rifampin and carbamazepine, which are strong inducers, while phenytoin is a moderate inducer. The Flockhart table also lists 33 CYP2C9 substrates, including warfarin and glipizide, which may interact with ivacaftor [26].

The current literature recommends ETI dose modifications for specific adverse events, e.g., anxiety and depression. The OpenVigil database was used to identify adverse events of anxiety and depression and investigated whether such dose modifications, if any, had been made. The OpenVigil database was also used to determine if dose modification of ETI was carried out for potential drug–drug interactions (CYP 3A 4/5 inhibitors and inducers) of interest.

## 5. Results

The 2022/2023 CF data registries reported that 3798 people with CF were enrolled in Australia (2023), 4445 people with CF in Canada (2022), and 33,288 people with CF in the U.S. (2023) [27,28,29]. Across the three countries, between 50% and 70% of people with CF were taking ETI, 67% (*n* = 2530) in Australia, 51% (*n* = 2272) in Canada and 70% (*n* = 23,350) in the U.S., respectively.

During the study period, 10,628 entries of adverse events associated with ETI use had been reported on the databases (U.S. = 10,503; Canada = 80; Australia = 45) (Table 1). Most adverse events were reported in people with CF aged 18 to 64 years in the three databases, with more adverse events reported in females in the U.S. and Canadian databases.

The adverse events were reported in all 27 SOCs, with the top ten being gastro-intestinal, general disorders, injury, infections, investigations, nervous system disorders, psychiatric disorders, respiratory, skin and subcutaneous disorders, and surgical procedures (Table 2). The U.S. database reported more infections and investigations (11.4%) compared to Australia (5.6%) and Canada (7.1%). More psychiatric adverse events were reported in Australia (15.6%) and Canada (15.3%) compared to the U.S. (7.4%). The number of adverse events reported in each database was much greater than the actual number of reports, as most of the reports included more than one adverse event associated with ETI. The three most reported adverse events within the top 14 SOC are shown in Supplementary Materials.

The OpenVigil database reported 429 incidents of anxiety and depression (Table 3). These included 188 reports of anxiety, 136 reports of depression, and 105 reports of combined anxiety and depression. Of these, 118 (28%) resulted in changes in the ETI dose, including switching morning and night doses and reducing the dose (for example, one ETI tablet in the morning and one ivacaftor tablet at night). Antidepressants were listed as concomitant drugs in forty-three (10%) reports at the time they were submitted. Some of the reports of anxiety and depression also included other SOC adverse events such as respiratory and gastrointestinal.

Within the FAERS database, ciprofloxacin (*n* = 183) was the most common co-prescribed CYP3A4/5 inhibitor, followed by the azole antifungals (*n* = 105) (Table 4). The most common co-prescribed CYP3A4/5 inducer was prednisone/prednisolone (*n* = 241). The co-prescription of drug substrates of CYP2C9 varied between different drug classes, the most common being non-steroidal anti-inflammatory drugs (NSAIDs) (*n* = 190). Other CYP2C9 drug substrates included amitriptyline, irbesartan, losartan, fluoxetine, venlafaxine, rosuvastatin, and warfarin.

Data from the OpenVigil database showed the outcome to ETI doses when combinations of ETI and CYP3A4/5 inhibitors were co-prescribed (Table 5). Of the 57 people with CF who took ETI with one of the azole antifungals, 34 (60%) had their ETI dose modified. It was the highest and lowest for posaconazole-ETI (69%) and fluconazole-ETI (50%) combinations, respectively.

## 6. Discussion

This study was the first to compare spontaneous adverse events of ETI in three international databases of pharmacovigilance. Integrating and comparing adverse event reports from various databases enhances the analyses by expanding the sample size, diversifying data representation, and boosting the statistical power to identify associations.

The most common adverse events reported in the manufacturer’s clinical trials according to SOC categories included the following: (1) infections and infestations, (2) nervous system disorders, (3) respiratory disorders, (4) gastrointestinal disorders, (5) skin and subcutaneous disorders, and (6) investigations [30]. Common adverse events, which were reported in the manufacturer’s clinical trials and stated in the product information, were also reported in the pharmacovigilance databases. Other less common adverse events such as increase in blood pressure has also been stated in the product information and reported in the databases. However, the product information does not list all the uncommon, rare, and ultra rare adverse events that may have potentially been reported. Therefore, the real-world reporting of adverse events can highlight uncommon and rare adverse events. Our findings show a wide range of adverse events reported across all 27 SOCs, in comparison to what was reported in clinical trials and systematic reviews [10,11,12]. Notably, this study has highlighted several psychiatric adverse events (7 to 15% of all SOCs) reported in these databases.

Another key finding was the identification of commonly used concomitant drugs, not captured in clinical trials, which may have potentially contributed to drug–drug interactions. As people with CF’s life expectancy increases, they will be at risk of developing comorbidities associated with ageing, adding to their drug therapy burden and increasing their risk of drug–drug interactions. This study has demonstrated that people with CF were co-prescribed a range of drugs with potential drug interactions. Therefore, it is important to be aware of this significant risk and develop strategies to mitigate the risk in people with CF experiencing polypharmacy.

As the use of ETI has increased since 2019, a growing number of adverse events have been reported in various SOCs. Real world data provides a broader view of drug safety across specific populations with comorbidities. The majority of adverse events were reported in the age group of 18 to 64 years, as ETI was initially available for people with CF aged 12 and older and uptake of ETI was rapid in those aged 18 and older. Furthermore, more female patients than males reported adverse events. This is consistent with what has been reported in the general population, with women often under-represented in clinical trials [9].

Although people with CF may have psychiatric comorbidities, the psychiatric adverse events which have been associated with ETI as reported in the real world is significant [16,17]. These psychiatric adverse events such as anxiety and depression can significantly impact quality of life. Analysis of the reports of anxiety and depression available in the OpenVigil database revealed that modifications in ETI doses had been made in most instances. In some circumstances, more than one dose modification was made. However, it was not possible to determine when these dose modifications were implemented, nor whether they were prompted by the reported psychiatric adverse events. It was worth noting that several of these reports contained concomitant antidepressants (most common were selective serotonin reuptake inhibitors), but it was not possible to determine when these drugs were started or their respective doses. While not conclusive, our observation suggests that psychiatric adverse events may have played a role in some of the observed dosage adjustments which had been reported in two previous studies [31,32]. Both studies had demonstrated that some people with CF, who do not tolerate the full dose of ETI as a result of psychiatric adverse events, may require individualised dose adjustments to minimise adverse events to enable them to continue CFTR modulator therapy [31,32].

Several concomitant drugs with potential interactions with ETI were identified from the FAERS database. Ciprofloxacin was the most frequently prescribed moderate inhibitor of CYP 3A4/5. Prednisone and prednisolone, considered weak inducers, were also commonly co-prescribed with ETI in this database. However, these potential drug interactions were not considered significant. On the other hand, co-prescribing of strong CYP3A4/5 inhibitors such as azole antifungals (e.g., itraconazole and voriconazole) and strong inducers of CYP3A4/5, such as rifampin and carbamazepine, with ETI, warrants careful consideration, as these combinations may not produce the desired therapeutic benefit for people with CF. The OpenVigil database revealed a significant number of instances in which ETI doses were modified during co-administration with CYP3A4/5 inhibitors. However, it was unknown when these dose modifications were initiated. Of concern were cases where dose modifications were not made and whether this could have potentially contributed to the adverse event(s) reported. The identification of adverse events possibly involving drug interactions warrants further exploration; however, causality cannot be inferred from the available FAERS data due to inherent reporting limitations.

As a review of reported adverse events and drug interactions to regulatory authorities, there are several limitations to this study. Many reports included more than one adverse event which would have potentially confounded the analyses. The reports may have been completed by healthcare professionals and/or consumers, which affects the reliability of the data. The databases were limited by the amount of information provided and the level of detail provided for each adverse event varied. Some adverse events included extensive information, such as patient demographics and all concomitant drugs, while others only included information about the adverse event.

Another limitation is that this study may have included duplicate reports, as deduplication of the data was not possible due to different time points, potentially leading to an overestimation of some adverse events. Disproportionality analysis of the data was not able to be completed due to lack of deduplication of reports. Also, the lack of the total number of people with CF taking ETI over the study period made it difficult to determine the significance of the risks of the reported adverse events. Furthermore, data on patient co-morbidities, drug doses, when drugs were started or stopped, and if patients were taking the concomitant drugs at the time of the adverse event(s) being reported were not available in the databases. Consequently, it was not possible to determine whether the concomitant drugs may have contributed to the adverse events as a direct result of drug interactions. Generally, there is an underreporting of adverse events and a level of selective reporting of adverse events for new drugs. Therefore, it was not possible to draw firm conclusions on the incidence and causality of these adverse events of ETI, as reported in these databases. More studies are needed to determine the associations between adverse events and ETI, and the impact of potential drug–drug interactions.

## 7. Conclusions

This descriptive study examined the prevalence and types of adverse events associated with ETI in the real world, with a particular focus on potential drug–drug interactions. Adverse events were reported across all 27 system organ classes, reflecting a broad range of adverse outcomes. Psychiatric adverse events have been previously documented in the literature, and this study confirmed their prevalence. Furthermore, the co-prescribing of ETI with CYP3A/45 inhibitors and inducers, despite being well-known interaction risks, highlights a critical area for clinical vigilance. Our preliminary findings emphasise the need for further research to elucidate the underlying mechanisms of these adverse events and to develop evidence-based strategies for mitigating risks associated with ETI therapy and its concomitant use with interacting drugs. Ensuring safe and effective use of ETI is a priority in optimising health outcomes for people with CF.

## Figures and Tables

**Table 1:** Patient demographics.

	Australia		Canada		U.S.	
Number of adverse events reports	*n* = 45		*n* = 80		*n* = 10,503	
Patient demographics						
Gender						
No. Males	18	(40%)	34	(43%)	4029	(38%)
No. Females	17	(38%)	41	(51%)	5333	(51%)
Unspecified	10	(22%)	5	(6%)	1141	(11%)
Age						
By age 0 to 11 yrs	7	(16%)	10	(12%)	429	(4%)
12 to 17 yrs	6	(13%)	12	(15%)	944	(9%)
18 to 64 yrs	19	(42%)	51	(64%)	3571	(34%)
65 to 85 yrs	1	(2%)	1	(1%)	134	(1%)
>85 yrs					1	
Unspecified	12	(27%)	6	(8%)	5424	(52%)

**Table 2:** Prevalence of adverse events in the three databases.

	Australia		Canada		U.S.	
Number of adverse events by SOC	*n* = 179		*n* = 254		*n* = 19,595	
Blood and lymphatic system disorders	2	(1.1%)	-		78	(0.4%)
Cardiac disorders	2	(1.1%)	1	(0.4%)	109	(0.6%)
Congenital, familial, and genetic disorders	-		2	(0.8%)	222	(1.1%)
Ear and labyrinth disorders	1	(0.6%)	-		89	(0.5%)
Endocrine disorders	-		-		27	(0.1%)
Eye disorders	2	(1.1%)	-		349	(1.8%)
Gastrointestinal disorders	24	(13.4%)	25	(9.8%)	2011	(10.3%)
General disorders and administration site conditions	16	(8.9%)	19	(7.5%)	1922	(9.8%)
Hepatobiliary disorders	3	(1.7%)	7	(2.8%)	403	(2.1%)
Immune system disorders	-		2	(0.8%)	149	(0.8%)
Infections and infestations	10	(5.6%)	18	(7.1%)	2242	(11.4%)
Injury, poisoning, and procedural complications	5	(2.8%)	11	(4.3%)	910	(4.6%)
Investigations	20	(11.2%)	45	(17.7%)	1803	(9.2%)
Metabolism and nutrition disorders	8	(4.5%)	5	(2.0%)	463	(2.4%)
Musculoskeletal and connective tissue disorders	4	(2.2%)	9	(3.5%)	466	(2.4%)
Neoplasms benign, malignant, and unspecified	1	(0.6%)	-		86	(0.4%)
Nervous system disorders	9	(5.0%)	12	(4.7%)	1511	(7.7%)
Pregnancy, puerperium, and perinatal conditions	3	(1.7%)	6	(2.4%)	176	(0.9%)
Product issues	-		-		32	(0.2%)
Psychiatric disorders	28	(15.6%)	39	(15.3%)	1451	(7.4%)
Renal and urinary disorders	2	(1.1%)	3	(1.2%)	211	(1.1%)
Reproductive system and breast disorders	-		1	(0.4%)	198	(1.0%)
Respiratory, thoracic, and mediastinal disorders	14	(7.8%)	7	(2.8%)	1955	(10
Skin and subcutaneous tissue disorders	17	(9.5%)	30	(11.8%)	1251	(6.4%)
Social circumstances	1	(0.6%)	-		79	(0.4%)
Surgical and medical procedures	5	(2.8%)	11	(4.3%)	1286	(6.6%)
Vascular disorders	2	(1.1%)	1	(0.4%)	116	(0.6%)

Note: some categories do not have any adverse events reported.

**Table 3:** Prevalence of anxiety and depression in the OpenVigil database.

No. of Reports	Unknown Dose *	Dose Reduced/Switched ***	Standard Dose **	Dose Reduced/Switched ***
Anxiety (*n* = 188)	128 (68%)	30 (16%)	60 (32%)	23 (12%)
Depression (*n* = 136)	83 (61%)	17 (13%)	53 (39%)	15 (11%)
Anxiety and depression (*n* = 105)	57 (54%)	10 (10%)	48 (46%)	23 (22%)

* Dose of ETI used not specified in report. ** Standard dose of ETI specified in report. *** Dose modifications documented in reports with unknown or standard ETI dose.

**Table 4:** Prevalence of CYP 3A 4/5 inhibitors and inducers, and CYP 2C9 substrates co-prescribed in people with CF in the FAERS database.

Drug—CYP 3A 4/5 Inhibitor	Inhibitor Strength Level *	No. of Reports	Drug—CYP 3A 4/5 Inducer	Inducer Strength Level *	No. of Reports	Drug- Substrates CYP 2C9	No. of Reports
Atomoxetine	Weak	6	Carbamazepine	Strong	2	Amitriptyline	38
Ciprofloxacin	Moderate	183	Prednisolone/Prednisone	Weak	241	Celecoxib	24
Clarithromycin	Strong	37	Rifabutin	Weak	1	Diclofenac	17
Erythromycin	Moderate	17	Rifampin	Strong	7	Doxepin	2
Fluconazole	Moderate	41				Fluoxetine	86
Itraconazole	Strong	19				Glipizide	4
Ketoconazole	Strong	4				Ibuprofen	144
Posaconazole	Strong	24				Irbesartan	5
Voriconazole	Strong	17				Losartan	12
						Naproxen	29
						Rosuvastatin	15
						Torsemide	8
						Venlafaxine	31
						Warfarin	12

* Inhibitor/Inducer strength level classified by Flockhart drug interaction table or ETI product information.

**Table 5:** Modification of ETI dose with CYP 3A4/5 inhibitors from the OpenVigil database.

CYP 3A4/5 Inhibitor	No. of Reports Dose Modified		No. of Reports Dose not Changed		No. of Reports Dose Unknown	
Itraconazole (*n* = 16)	10	(63%)	1	(6%)	5	(31%)
Fluconazole (*n* = 14)	7	(50%)	1	(7%)	6	(43%)
Posaconazole (*n* = 16)	11	(69%)	3	(19%)	2	(12%)
Voriconazole (*n* = 11)	6	(55%)	1	(9%)	4	(36%)
Erythromycin (*n* = 4)	1	(25%)	1	(25%)	2	(50%)
Clarithromycin (*n* = 7)	1	(14%)	3	(43%)	3	(43%)

## Data Availability

No new data were created or analyzed in this study.

## Supplementary Materials

The following supporting information can be downloaded at: https://www.mdpi.com/article/10.3390/life15081256/s1, Table S1: Types and number of adverse events in the top 14 SOCs. The top three adverse events within each SOC are also shown.
