# Supplementary material for: Adverse Events and Drug Interactions Associated with Elexacaftor/Tezacaftor/Ivacaftor Treatment: A Descriptive Study Across Australian, Canadian, and American Adverse Event Databases
Source: Life (Basel). 2025 Aug 7;15(8):1256. doi: 10.3390/life15081256 (PMC12387162; doi:10.3390/life15081256)
Supplement: Supplementary file 1 [file life-15-01256-s001.zip › life-3751426-supplementary.pdf]

Table S1: Types and number of adverse events in the top 14 SOCs. The top three adverse events within each SOC are also shown.

SOC	Australia	No AE	Canada	No AE	US	No AE
**Infections and infestations**		**10**		**18**		**2,242**
	Lower respiratory tract infection	2	Infective Pulmonary Exacerbation of Cystic Fibrosis	2	Infective Pulmonary Exacerbation of Cystic Fibrosis	651
	COVID-19	1	Respiratory tract infection	1	Influenza	244
	Ear infection	1	Epididymitis	1	Pneumonia	211
**Gastrointestinal disorders**		**24**		**25**		**2,011**
	Gastrointestinal disorder	6	Abdominal pain upper	2	Abdominal Pain Upper	419
	Diarrhoea	4	Nausea	1	Diarrhoea	348
	Abdominal distension	2	Gastroesophageal reflux disease	1	Nausea	287
**Respiratory, Thoracic**		**14**		**7**		**1,955**
	Asthma	2	Dyspnoea	1	Cough	489
	Dyspnoea	2	Sinus disorder	1	Productive Cough	458
	Cough	1	Productive cough	1	Dyspnoea	220
**General disorders**		**16**		**19**		**1,922**
	Illness	3	Crying	1	Fatigue	415
	Malaise	3	Drug interaction	1	Illness	182
	Crying	2	Pain	2	Chest Discomfort	163
**Investigations**		**20**		**45**		**1,803**
	Weight increased	7	Hepatic enzyme increased	5	Weight Increased	405
	Liver function tests increased	2	Ejection fraction decreased	1	Alanine Aminotransferase Increased	170
	Pulmonary function tests decreased	2	Aspartate aminotransferase increased	4	Hepatic Enzyme Increased	163
**Nervous system disorders**		**9**		**12**		**1,511**
	Brain fog	1	Headache	3	Headache	693
	Disturbance in attention	1	Ageusia	1	Dizziness	213
	Formication	1	Neurological symptom	1	Brain Fog	154
**Psychiatric disorders**		**28**		**39**		**1,451**
	Depression	5	Anger	2	Anxiety	435
	Suicidal ideation	5	Aggression	4	Depression	315
	Anxiety	4	Anxiety	5	Insomnia	258

**Skin and subcutaneous tissue disorders**		**17**		**30**		**1,251**
	Rash	10	Pruritus	4	Rash	525
	Rash pruritic	2	Rash	4	Acne	146
	Urticaria	2	Urticaria	2	Pruritus	131
**Musculoskeletal and connective tissue disorders**		**4**		**9**		**466**
	Arthralgia	1	Back pain	2	Arthralgia	121
	Arthritis	1	Arthralgia	1	Back Pain	80
	Back pain	1	Myalgia	1	Pain In Extremity	50
**Metabolism and Nutrition disorders**		**8**		**5**		**463**
	Dairy intolerance	2	Decreased appetite	2	Decreased Appetite	100
	Food intolerance	2	Abnormal weight gain	1	Increased Appetite	71
	Dehydration	1	Increased appetite	1	Dehydration	54
**Hepatobiliary disorders**		**3**		**7**		**403**
	Acute hepatic failure	1	Cholelithiasis	1	Drug-Induced Liver Injury	67
	Bile duct stone	1	Drug-induced liver injury	1	Liver Disorder	48
	Drug-induced liver injury	1	Hepatic cirrhosis	1	Cholelithiasis	42
**Eye disorders**		**2**				**349**
	Lacrimation	1			Dry Eye	72
	Increased visual impairment	1			Visual Impairment	50
					Vision Blurred	48
**Renal and Urinary disorders**		**2**		**3**		**211**
	Renal impairment	1	Micturition urgency	1	Nephrolithiasis	77
	Urinary retention	1	Bladder disorder	1	Chromaturia	31
			Immunoglobulin A nephropathy	1	Acute Kidney Injury	25
**Cardiac disorders**		**2**		**1**		**109**
	Palpitations	2	Cardiomyopathy	1	Palpitations	19
					Tachycardia	13
					Cardiac Disorder	10

SOC: System Organ Class; AE: Adverse Event.
